# Simultaneous detection of respiratory virus RNA on environmental surfaces in a university setting by a sensitive Surface 3-Step PCR platform

**DOI:** 10.1038/s41598-025-22296-5

**Published:** 2025-11-03

**Authors:** Chiara Orlandi, Giulia Amagliani, Giorgio Brandi, Asja Conti, Giuditta Fiorella Schiavano, Anna Casabianca

**Affiliations:** 1 Department of Biomolecular Sciences, Section of Biochemistry and Biotechnology, University of Urbino Carlo Bo, Fano (PU), 61032 Italy; 2 Laboratorio Covid, University of Urbino Carlo Bo, Fano (PU), 61032 Italy; 3 Department of Biomolecular Sciences, Unit of Hygiene, University of Urbino Carlo Bo, Urbino (PU), 61029 Italy; 4 Department of Humanities, University of Urbino Carlo Bo, Urbino (PU), 61029 Italy

**Keywords:** Environmental surfaces, Fomites, High-touch surface, Real-time RT-PCR multiplex assay, Respiratory viruses, University setting, Diseases, Health care, Microbiology

## Abstract

Most respiratory viruses like SARS-CoV-2 spread through aerosols and fomites, remaining viable in the air and on surfaces. The present study aims to detect simultaneously by a multiplex molecular approach (Surface 3-step PCR platform), the presence of the three major co-circulating respiratory viruses (SARS-CoV-2, Flu A/B and RSV A/B) from inert surface samples in non-healthcare environments on a university setting in Central Italy. In total, 400 environmental surface swabs were collected during the study period in a three time point longitudinal program (T1, the end of the first semester: November-December 2023, weeks 48–49; T2, the extraordinary exam session: January 2024, weeks 2–4; T3, the start of the 2nd semester: February 2024, weeks 8–9) among which 62 (16%) were positive for viral RNA and with the positive rate that dropped from 20% (25/125) to 8% (10/130). The frequency of environmental contamination was higher in small classrooms (30/135, 22%) than in medium (15/105, 14%) and large (13/115, 11%) ones. Here, we describe the use of a novel rapid and sensitive combined multistep molecular platform involving two process controls, one synthetic RNA added directly to the sample and one endogenous human, able to detect low copy numbers of viral RNA targets in high-touch surfaces, with high sensitivity (98% of valid results). This study shows the potential as an effective solution to apply targeted interventions to prevent the spread of the airborne infections within the university community.

**Supplementary Information:**

The online version contains supplementary material available at 10.1038/s41598-025-22296-5.

## Introduction

Viruses are the most common cause of respiratory infections, which often lead to comorbidity and mortality in children and adults^1^ and pose significant public health challenges worldwide.

The early detection of respiratory viruses is essential for reducing the risks associated with infection, nosocomial transmission, and inappropriate treatment^2^.

Several different respiratory viruses can circulate simultaneously and can potentially infect the same host. Factors like globalization and intensive urbanization have fostered viral transmission leading to catastrophes like the coronavirus disease 2019 (COVID-19) pandemic.

Severe acute respiratory syndrome coronavirus (SARS-CoV-2), influenza virus (Flu) and respiratory syncytial virus (RSV) are major pathogens that primarily target the human respiratory system^3^. SARS-CoV-2 causes fairly nonspecific symptoms, and the onset of disease coincides with the active Flu and RSV season^4^.

Influenza remains a major public health threat, causing significant morbidity and mortality, especially among vulnerable populations. The potential for influenza viruses to mutate and reassort can lead to the emergence of new strains. About these, Influenza A viruses that infect humans mainly consist of two strains (H1N1 and H3N2) and during the flu season can exert a substantial burden on healthcare systems, particularly during concomitant other respiratory infections, such as COVID-19^5^.

RSV is the pathogen that affects approximately 60–70% of all children before one year of age, and nearly all children are infected before the age of two^6^ and usually causes mild, cold-like symptoms. In Western countries, mortality due to an RSV infection is rare; however, RSV infections in infants and young children have a substantial impact on the healthcare system, require hospitalization^7^ and furthermore can significantly disrupt parental and grandparental activities, leading to work absences and reduced productivity^8^.

Respiratory viruses primarily transmit via three main routes. Droplets or aerosols (“infectious respiratory particles”, IRPs^9^ formed in the respiratory tract of a diseased person can be ejected via talking, sneezing, breathing, or coughing to directly enter the recipient^10^. Physical contact with the surface with a previously deposited droplet or inanimate objects (fomite) can serve as a source of infection spreading. Fomites can also be called passive vectors. The third route of transmission is particularly airborne transmission^11^.

More recently, the monitoring of viruses in the environment, including fomites, wastewater, etc., has emerged as a useful tool for to track their spread and for community surveillance aimed at the epidemiological control of communicable diseases (SARS CoV-2, influenza, Mpox)^12–15^. Several studies have investigated the presence of viral RNA in environmental surfaces, although with a focus on healthcare settings^16,17^. A comprehensive review^18^ revealed the presence of SARS-CoV-2 contamination on various surfaces across different types of facilities; in particular, the study found that approximately 17.7% of samples collected from hospital settings and 10.1% of samples from non-hospital settings tested positive for SARS-CoV-2 RNA. This indicates that significant levels of surface contamination with the virus, primarily on surfaces made of plastic, glass, paper, metal and stainless steel^19^, are prevalent in both healthcare and non-healthcare environments. But hospitals have already standardized disinfection measures, compared with other settings, e.g., community and household^20,21^.

It was pointed out that most viruses from the respiratory tract (i.e.: corona, coxsackie, influenza, SARS or rhino virus) could persist on surfaces for a few days, being a potential source of transmission, if surface disinfection is not preventively performed. The viruses survived on some of the surfaces for up to 6 days post-contamination, but not after 9 days and survived longer on nonporous surfaces than on porous ones^22^.

This suggests that frequently touched surfaces in hospitals and public places could be significant sources of transmission for SARS-CoV-2, influenza virus and other viruses that could survive on environmental surfaces for extended periods, sometimes up to months^23,24^. Therefore, prolonged potential transmission of virus might occur via contact or fomite.

Once again, even if non-clinical settings, such as university setting, have received less attention from studies investigating environmental surface contamination, the risk of fomite-mediated transmission in such environments cannot be understated, as residents are less protected and surfaces are not disinfected as frequently as in hospital settings^25^.

Thus, surface investigation performed simultaneously by molecular based-assays for the most important respiratory viruses may prove useful both for the evaluation of contaminated fomites as a source of viruses that may cause infections in susceptible individuals and as a source of information regarding the dynamics of infectious disease in the population.

Recently, our group^26^ published the results of a pilot study concerning the environmental monitoring of SARS-CoV-2 on high-touch surfaces in a university setting, detecting the presence of SARS CoV-2 RNA.

The presence of viral genetic material on the surfaces reveals the transit and contact of infected individuals. Even if surface testing is a complement to preventive measures and environmental monitoring plans, it remains useful as part of the risk assessment to also ensure attending people’s safety.

Due to the high level of shared living situations and services (classrooms and academic buildings, bathrooms and kitchens) and high social activity, university students have substantially more contacts than the general population^27^. Therefore, although generally at lower risk for severe disease than older age groups, infected young adults have more opportunities to transmit infections to others in their communities^28^. Furthermore, young adults show less adherence to isolation recommendations^27^ and only a very little number of them with acute respiratory infection stay at home while ill^29^, thus contributing to infection circulation.

In the post-pandemic era, a few studies investigated the effectiveness of different measures to mitigate transmission of COVID-19 and other airborne infections among university students, faculty members, and staff^30,31^. Besides periodic PCR-based or antigen testing for individuals, social distancing and use of protective equipment (i.e., face masks), essential in particular epidemiologic situations, the usefulness of nonpharmaceutical interventions as strategies to mitigate transmission of COVID-19 and other respiratory diseases has been strongly supported^28^. Among them, environmental prevention measures, like the installation of ultraviolet (UV)-based air disinfection systems and the environmental monitoring of viral nucleic acids in indoor surfaces through high-throughput molecular assays, appear particularly promising. Indeed, environmental molecular testing offers the advantage of providing a cost-effective and non-invasive approach to monitor virus spread and to implement an early warning system. However, the selection of an appropriate detection method, able to track only minimal amounts of viral nucleic acids and to recognize possible amplification inhibition (false negative results), is a key element.

The monitoring of environmental viral genetic material on fomites could support public health prevention strategies in the academic and, in general, in work environments. For this reason, the purpose of our research was to evaluate by a sensitive multiplex real-time PCR platform the spread and intensity of Flu viruses, RSV and SARS-CoV-2 circulating during the 2023–2024 autumn-winter season on environmental surfaces in a non-healthcare public place, such as a university setting frequented by students and staff involved in the academy activity.

The environmental monitoring at university, besides being a non-invasive approach to assess viral RNA presence, may provide both information for targeted prevention measures and an early warning system in case of infection cluster or outbreak.

## Materials and methods

### Sampling locations and methods applied

The study began in November-December 2023 (weeks 48–49, i.e., the end of 1 st semester; T1) and continued in January 2024 (weeks 2–4, i.e., the extraordinary exam session; T2) ending in February 2024 (weeks 8–9, i.e., the start of 2nd semester lessons; T3). Environmental surface swabs (*n* = 400) were collected as described in Casabianca et al.^26^ in different surface types and locations of the University of Urbino Carlo Bo including classrooms with different student capacities (S: small < 40, M: medium < 100, L: large > 100 students) and common areas such as bathrooms and recreation areas, with particular regard to student’s desks, teacher’s workstation, handles, light switches, study and dining tables, toilets, vending machine push-button panel, chair armrests (Tables 1 and 2). As for a typical environmental monitoring program, samplings were directed to identify high-touch surfaces and fomites in university settings in indoor areas exposed to human crowding or frequently touched by hands, which included shared workstations (mouses and keyboards), computer accessories, doorknobs, tabletops, and vending machines.

The environmental samples were collected using a swab with a synthetic tip and a plastic shaft soaked in DNAse/RNAse-free water. The recommended swab surface area of 100 cm^2^ was sampled by swabbing the entire surface horizontally and vertically, rotating the swab throughout^32^. Each swab was then placed in a tube containing 500 µl of a guanidine solution (viral transport medium, VTM) which inactivates and stabilizes the viral nucleic acids (Zymo Research, Irvine, CA, USA or Zybio Inc Chongqing, China, based on availability). The samples were kept at −20° C until the nucleic acid extraction step.

**Table 1:** Number of surface samples, based on classroom capacity (Small < 40, Medium < 100, Large > 100 students) and common areas (such as bathrooms and recreation areas), at the various time points and overall.

	T1	T2	T3	Overall
Small	42 (33%)	53 (38%)	42 (31%)	137 (34%)
Medium	37 (29%)	26 (19%)	44 (33%)	107 (27%)
Large	33 (26%)	42 (30%)	42 (31%)	117 (29%)
Common areas	15 (12%)	18 (13%)	6 (4%)	39 (10%)
Total	127 (100%)	139 (100%)	134 (100%)	400 (100%)

**Table 2:** Number of positive samples based on sampling location, at the various time points and overall.

	T1	T2	T3	Overall
Student’s desks	18/83	14/86	7/102	39/271
Teacher’s workstation	3/22	12/29	3/19	18/70
Handles, light switches (classrooms)	1/2	0/1	-	1/3
Study and dining tables	3/10	1/11	0/1	4/22
Toilets	0/4	-	-	0/4
Vending machine push-button panel	0/1	0/6	0/4	0/11
Chair armrests	0/3	0/3	0/4	0/11
Total	25/125	27/136	10/130	62/391

### RNA extraction

Nucleic acids from samples were extracted using Virus Nucleic Acid Isolation Kit (PureDireX) starting from 250 µl of VTM and following the manufacturer’s instructions. To evaluate RNA extraction efficiency and identify the presence of PCR inhibitors, 0.5 µl (i.e.; 1/100 of the elution volume) of a synthetic RNA process control (Internal Process Control, IPC) was added to each sample. Purified RNA was stored at −80 °C until analysis.

### Surface 3-step PCR

Each sample was analysed by means of a multi-step amplification approach, named Surface 3-step PCR, which is based on the sequential use of three different real-time PCR assays: *(1)* the CE-IVD certified SARS-CoV-2 Surface Kit for the detection of SARS-CoV-2 (RdRp gene) and the IPC (Diatheva, Italy); *(2)* the CE-IVD certified COVID-FLU-RSV All-in-One RT PCR (Diatheva) for the simultaneous detection of respiratory viruses, targeting their specific regions (ORF1b/N genes for SARS-CoV-2, M1/NS2 genes for Flu A/B, M gene for RSV A/B) and the human RNase P gene as endogenous control; *(3)* an in house developed SYBR Green qPCR assay, used for the quantification of the Rpp40 single copy gene (part of the human RNase P gene family), to provide a further endogenous control for human DNA^33^. All analyses were carried out on the QuantStudio 5 Real-Time PCR Systems (Thermo Fisher Inc., CA, USA) in a 10 µL reaction containing 2.5 µL nucleic acid extracted. The cDNA synthesis and thermal cycle conditions for each PCR step are detailed in Supplementary Table S1.

The limit of detection (LOD) for SARS-CoV-2 target corresponds to 3.83 copies/PCR, for FluA it is 16.59 copies/PCR, for Flu B it is 2.39 copies/PCR, for RSV it is 1.28 copies/PCR. The analytical sensitivity of the COVID-FLU-RSV All-in-One RT PCR kit has been previously determined using serial dilutions of synthetic RNA controls for SARS-CoV-2, Influenza A, Influenza B and RSV (subtype A) and analyzed by Probit analysis (*p* = 0.005, 95% CI, as declared in the manufacturer’s documentation^34^. The LOD for Rpp40 target corresponds to 1 copy/PCR and has been determined using serial dilutions of the specific plasmid containing the fragment of the Rpp40 gene.

### Interpretation of results

**Fig. 1:**
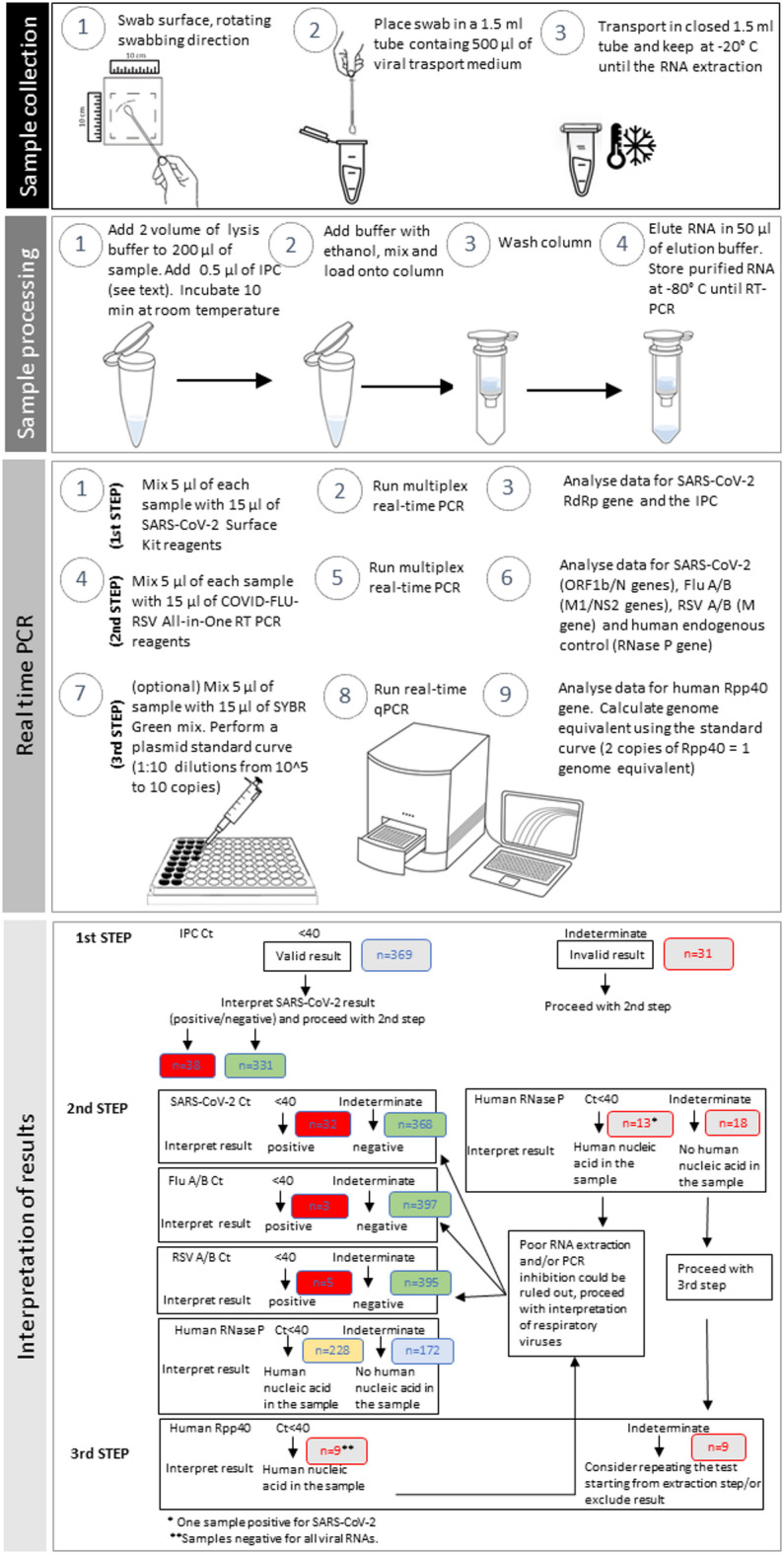
Workflow of the Surface 3-step PCR for SARS-CoV-2, Flu A/B and RSV A/B RNA detection. The environmental samples are collected using a swab with a synthetic tip and a plastic shaft soaked in DNAse/RNAse-free water, sampling a surface area of 100 cm^2^. A spin column-based method is used for the extraction and purification of nucleic acids. The processed sample is then mixed with PCR reagents and subjected to the multiplex real-time PCR assays using specific primers and fluorogenic probes (1st and 2nd steps) and to the SYBR Green qPCR (3rd step), if necessary. The viral RNA presence can be determined by interpretation of the PCR Ct (cycle threshold) value. The numbers in the bottom panel refer to the interpretation of results for 400 environmental samples.

The workflow of the Surface 3-step PCR for SARS-CoV-2, Flu A/B and RSV A/B RNA detection and data analysis is reported in Fig. 1. Results were interpreted as follows: in the first step, samples with amplification signals of RdRp, with or without IPC, were assigned as SARS-CoV-2 positive; samples testing negative for RdRp, having the IPC Ct > 40 were assigned as invalid. In the second step, samples with Ct ≤ 40 for each viral target, with or without RNase P, were assigned as positive; samples testing negative for viral targets having the RNaseP Ct > 40 were assigned as invalid. In the third step, samples with the Rpp40 Ct > 40 were assigned as invalid and excluded from the prevalence calculation.

### Statistical analysis

Continuous data are given as means and standard deviations (SD) or medians and interquartile ranges [IQR], and categorical data are given as counts and percentages. After data screening for normal distribution, the Kruskal-Wallis test followed by Dunn’s multiple comparisons post-test or ANOVA, was used for comparisons over time. The Mann Whitney test was used for comparison between two data sets. P-values lower than 0.05 were considered statistically significant. The analyses and graphs were performed using GraphPad Prism (version 8.4.2, GraphPad Software, San Diego, CA, USA).

## Results

### Amplification analysis of the internal process control and human targets

To assess the reliability of the Surface 3-step PCR method, three different extraction/amplification control systems have been used: the addition of the synthetic RNA oligonucleotide IPC to the samples during extraction, amplified in the first step; the amplification of the human RNaseP gene in the second step as endogenous control for human DNA; the amplification of the Rpp40 single copy gene in the third step as additional endogenous control.

**Fig. 2:**
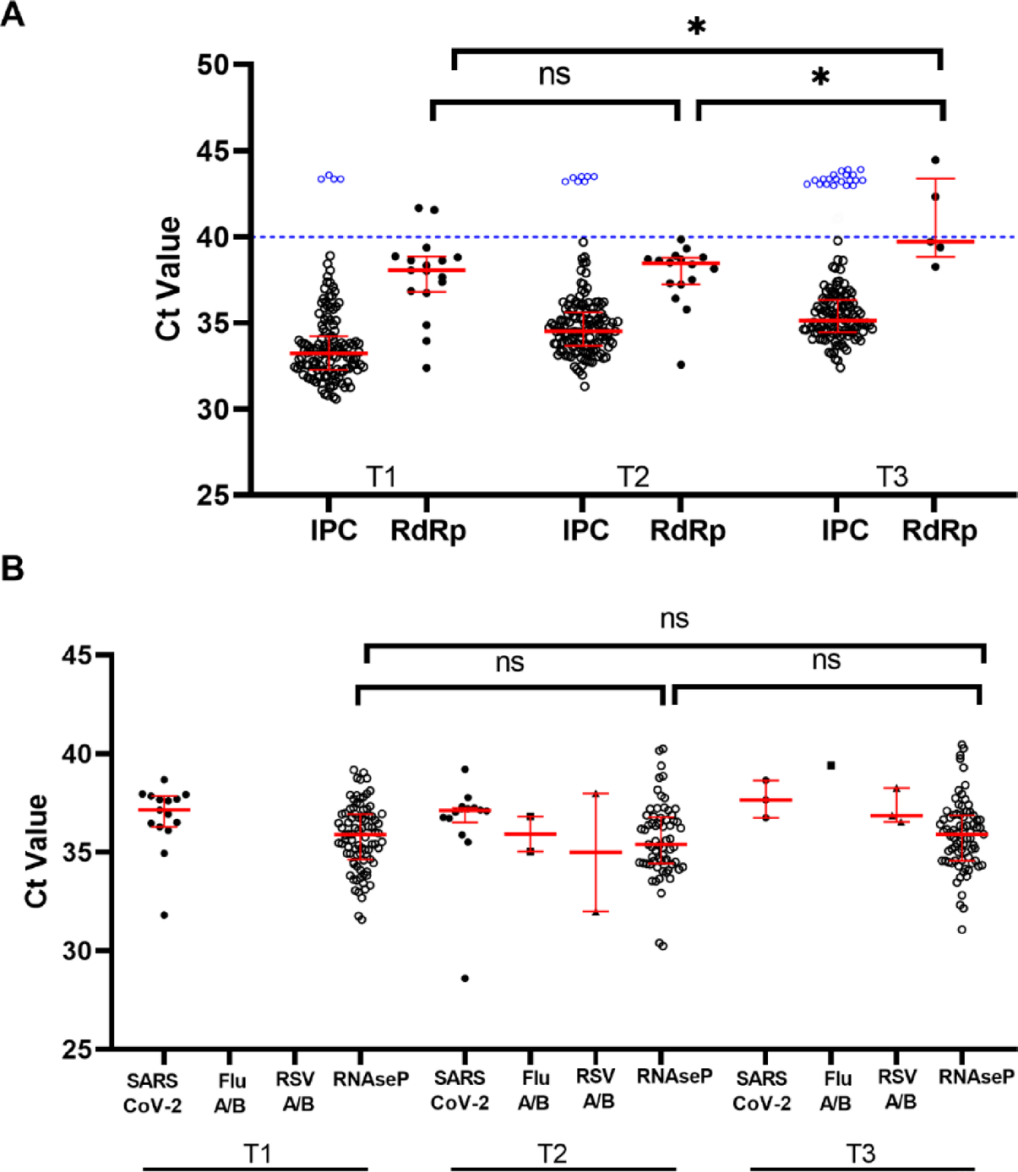
**(A)** Results from the first-step PCR in 400 environmental samples at the various time points (T1: November-December 2023, weeks 48–49; T2: January 2024, weeks 2–4; T3: February 2024, weeks 8–9); 4, 7 and 20 samples with an invalid result (Ct value > 40) for the Internal Process Control (IPC) are represented as blue circles above the dashed line. Among 369 valid samples, 17, 16 and 5 gave a positive amplification for RdRp gene of SARS-CoV-2 RNA. **(B)** Results from the second-step PCR in 400 environmental samples at the various time points. Red lines represent the median and 25th to 75th percentiles. Statistical significance was assessed using the ANOVA test, *p < 0.05; ns: not significant.

The application of the first step allowed us to obtain valid results from the 92.2% of samples (369/400), in which the IPC was correctly amplified with a Ct ≤ 40 (according to the supplier’s indications) with a median Ct value of 34.48 [IQR: 33.29–35.73], (Fig. S1A, Fig. 2A, and Supplementary Table S2). The remaining 7.8% (31/400, 4 at T1, 7 at T2 and 20 at T3) were assigned as “invalid test”, due to the failure of IPC amplification, or Ct > 40. In the second step of the Surface 3-step PCR all the 400 samples were analysed for the presence of human DNA, detected through the amplification of a RNaseP specific sequence. In the 42% (13/31) of the samples with an invalid result at first step, the amplification of the endogenous control with a median Ct value/PCR of 36.63 [IQR: 35.43–37.13] confirmed the recovery of human nucleic acid, thus allowing to rule out poor RNA extraction and/or PCR inhibition in those samples. The remaining 58% (18/31) of invalid samples were subjected to the third step of the Surface 3-step PCR, checking for the presence of an alternative human DNA fragment (Rpp40), a single copy gene part of the human RNase P gene family. This approach provided a further control exploitable to assess the amplifiability of human DNA and to quantify its presence. Indeed, by means of a standard curve with a high PCR efficiency of 100% and 2 copies as limit of quantification, the Rpp40 also allowed to obtain the number of the genome equivalents per sample. Half of the samples (9/18) had a quantifiable Rpp40 content with a median Ct value of 31.88 [IQR: 31.22–33.20] corresponding to a median 59 [IQR:24–95] genome equivalents per sample, while the other half tested negative, thus remained invalid. Therefore, these 9 samples (2.3% of the total, 2 at T1, 3 at T2 and 4 at T3) were excluded from the analysis.

In Supplementary Table S3 are reported the Ct values of PCR controls. The positive PCR control always gave an amplification signal (median [IQR] 28.96 [28.88–29.65] for IPC, 32.30 [32.01–32.91] and 22.21 [21.97–23.01] for RdRp and ORF1b/N of SARS CoV-2 respectively, 26.41 [26.18–27.01] for Flu A/B, 25.97 [25.62–26.03] for RSV A/B (M) and 21.42 [20.87–22.09] for human RNase P. The negative PCR control always gave no amplification signal for both IPC and viral or human genes, confirming the accuracy of the PCR experimental procedure.

### Temporal change of the detection of the SARS-CoV-2, influenza A/B and respiratory syncytial viruses A/B

**Fig. 3:**
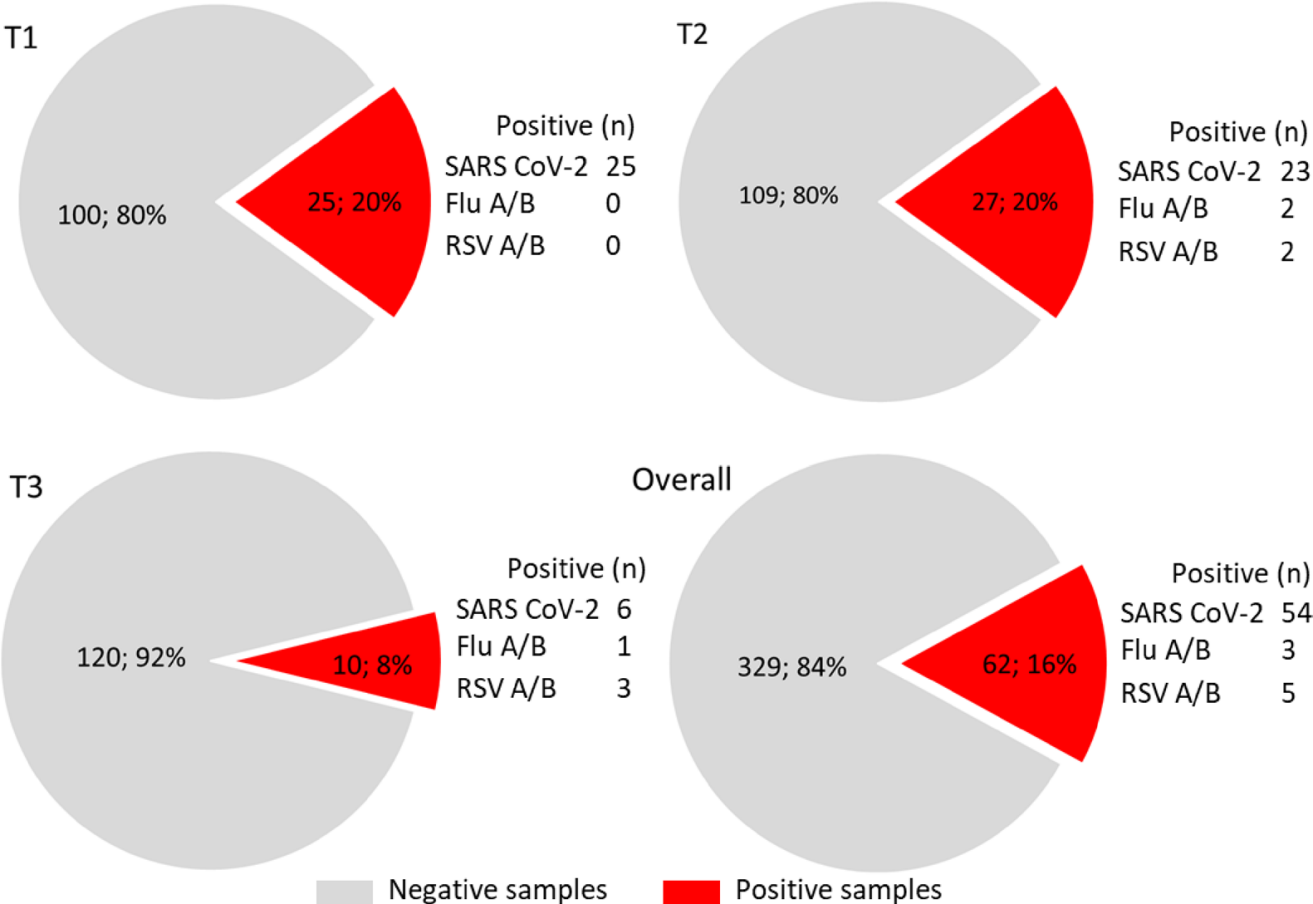
Positivity rate for the respiratory viruses tested on surface samples at various time points (T1: November-December 2023, weeks 48–49; T2: January 2024, weeks 2–4; T3: February 2024, weeks 8–9).

During the autumn-winter 2023–2024, 400 environmental samples were collected at November-December 2023, weeks 48–49 (T1), January 2024, weeks 2–4 (T2), February 2024, weeks 8–9 (T3) at the University of Urbino Carlo Bo and subjected to the Surface 3-step PCR for SARS-CoV-2, Flu A/B and RSV A/B RNA detection. Overall, 62 (16%) surface samples resulted positive for the RNA of the respiratory viruses tested. Of these positive samples, 25 (20%) were collected at the T1, 27 (20%) at T2 and 10 (8%) at T3 (Fig. S1, Figs. 2 and 3, Table S2).

In total, 54 samples tested positive for SARS-CoV-2, corresponding to the 14% of the valid samples. Of these samples, 25 (20%) were detected at T1, whereas 23 (17%) and 6 (5%) at the T2 and at the T3, respectively. Concerning Flu A/B, 3 (0.8%) samples tested positive during the three time periods, with no positive samples at T1, 2 (1.5%) at T2 and 1 (0.8%) at T3. Finally, RSV A/B RNAs were detected in 5 (1.3%) samples distributed as 2 (1.5%) at T2 and 3 (2.3%) at T3 (Fig. 3). The Ct values were analyzed to evaluate possible variations of viral targets over times. An increasing trend in Ct values for ORF1b/N (SARS CoV-2), M1/NS2 (Flu A/B), M (RSV A/B) was found from T1 to T3, reaching statistical significance for the SARS-CoV-2 RdRp (*p* = 0.0199) (Fig. 2).

### PCR concordance among the different SARS-CoV-2 targets

Among the 54 SARS CoV-2 positive samples, 22 (40.7%) were positive only in the first assay (target gene: RdRp; median [IQR] Ct 38.61 [37.93–39.93-01]), 16 (29.6%) were positive only in the second assay (target genes: ORF1b/N; median [IQR] Ct 37.47 [36.86–37.96]), while 16 (29.6%) were coherently detected by both assays (37.96 [36.02–39.23] and 36.84 [35.60–37.19] for RdRp and ORF1b/N, respectively) (Supplementary Table S4). For each method, discordant samples showed higher median Ct values than for concordant samples, reaching statistical significance for the ORF1b/N-based assay (*p* = 0.0077).

### Room size

Overall, our results suggested an effect on positivity rates linked to classroom capacity, with the smaller having a higher prevalence of positive samples (Table 3). A similar distribution was evenly emphasized when the positivity rates were calculated over the 62 positive samples; indeed, the highest percentage was found in small classrooms (30/62, 48%), less in medium (15/62, 24%) and in large classrooms (13/62, 21%) and only 7% in common spaces (4/62).

**Table 3:** Positivity rates for the all respiratory viruses (SARS-CoV-2, flu A/B and RSV A/B RNA) on 391 surface samples, based on classroom capacity (Small < 40, Medium < 100, Large > 100 students) and common areas.

Classroom capacity	Positivity rate (%)			
	T1	T2	T3	Overall^a^
Small	15/42 (36%)	14/52 (27%)	1/41 (2%)	30/135 (22%)
Medium	6/37 (16%)	6/26 (23%)	3/42 (7%)	15/105 (14%)
Large	1/31 (3%)	6/42 (14%)	6/42 (14%)	13/115 (11%)
Common areas	3/15 (20%)	1/16 (6%)	0/5 (0%)	4/36 (11%)

^a^ Invalid samples are excluded from the analysis (*n* = 9).

### Surface types

Viral RNA was detected in the samples obtained from student’s desks, teacher’s workstations, handles and light switches in the classrooms, study and dining tables. None of the samples from toilets, vending machine push-button panels or chair armrests tested positive (Table 2).

## Discussion

According to WHO^9^, ‘infectious respiratory particles’ (IRPs) are potentially infectious particles generated by individuals infected with a respiratory pathogen, during the infectious stage of the disease. The pathogen containing IRPs, along with water and respiratory secretions, carried by expired airflow, exit the infectious person’s mouth/nose through breathing, talking, singing, spitting, coughing or sneezing and enter the surrounding air. Besides IRPs, water and electrolytes, mucosalivary fluid may also contain various cell types (e.g., epithelial cells and cells of the immune system)^35^. Contaminated surfaces are thus created when IRPs expelled into the air settle on a surface, or when an infected person transfers infectious respiratory secretions by firstly touching their own mouth, nose or eyes and then touching a surface^36^. Infectious pathogens on the contaminated surfaces are then transferred to another person who touches that contaminated surface and then their own mouth, nose or eyes. This is commonly known as indirect contact transmission.

Environmental monitoring of respiratory viruses is thus essential for preventing and containing epidemics, although their detection at very low concentrations could sometimes be challenging. This kind of surveillance, if routinely performed, especially in seasonal periods in which respiratory virus circulation is of particular concern for public health, could be a useful tool to promptly recognise an increase of their presence and to identify spaces in which sanitation measures could be particularly needed.

Several authors^37–41^, including our research group^26^ recently described the high sensitivity offered by the application of molecular-based systems for the environmental detection of SARS-CoV-2 or other airborne pathogens in crowded public buildings. Molecular detection allows to shorten detection time, increase sensitivity, and circumvent drawbacks associated with conventional viral culture^42^. However, the reliability of molecular detection methods in environmental epidemiological surveillance is mainly based on proper assessment and correct use of process controls, especially if considering the well-known inhibitory effect of contaminants of environmental origin on the amplification reactions.

In this study, a new Surface 3-step PCR platform, introducing a novel approach suitable to assess result reliability, has been developed. This system included a first phase in which an Internal Process Control (IPC), a synthetic RNA oligonucleotide, was added to the environmental sample during lysis, to be co-extracted and co-amplified along with the viral target. Thus, the presence/absence of the IPC-specific amplification product allowed to evaluate RNA extraction efficiency and identify the possible presence of PCR inhibitors. In our work, 31/400 (7.8%) samples gave an invalid test result (IPC Ct > 40), probably due to low recovery in the nucleic acid extraction/isolation procedure or the presence of PCR inhibitors or a low PCR efficiency of IPC amplification. Concerning potential inhibitors in this kind of samples, the presence of detergents used for surface cleaning could be hypothesized. Additionally, the inhibitory effect of the sampling swab itself, depending on its composition, has been previously described^43^. The use of a sampling/transport medium containing suitable neutralizing agents could be a potential solution. However, the first step of the method applied in our work allowed to detect SARS-CoV-2 RNA in 9.5% of samples, with a decreasing trend during time intervals and a main prevalence on student desks.

The second step of our approach provided results about a panel of three main respiratory viruses (SARS-CoV-2, influenza virus Flu A/B and respiratory syncytial virus RSV A/B), along with human RNase P gene as endogenous control. Indeed, although the human RNase P gene is not strictly an endogenous control for such environmental samples, it demonstrates the presence or passage of people in the sampling locations and the elimination of human DNA vehiculated by mucosalivary fluids. Moreover, the usefulness of exogenous control is restricted to the monitoring of possible errors in nucleic acid extraction and amplification, but not during sampling and pre-PCR handling. In contrast, the importance of the RNase P target relies on its natural presence in the target environment, offering the opportunity to monitor the entire workflow and avoiding further sample manipulation^44^. In our study, the proper amplification of RNaseP allowed to include in the analysis additional 13 samples, which were previously assigned as invalid in step 1.

Our results highlighted a certain rate of disagreement between samples testing positive for SARS-CoV-2. The detection of SARS-CoV-2 RNA, based on different target sequences with respect to that of step 1 (ORF1b/N vs. RdRp), allowed to confirm the positivity of 16 samples, to detect 16 additional samples, but failed to identify 22 samples which tested positive in the first phase. A recent comparison highlighted that assays targeting the N gene were more specific than those targeting the RdRp^45^. Various real time RT-PCR assays are currently used worldwide, targeting different genes of the SARS-CoV-2, such as the envelope (E), ORF1ab/RNA-dependent RNA polymerase (RdRp), nucleocapsid (*N*), and spike (*S*) genes^46^. However, different RT-PCR assays would not be expected to have similar sensitivity, even when testing the same sample and several factors can affect the final Ct value such as thermocycling parameters and the optical threshold of detection^47^. As reported by Jeong et al.^48^, sensitivity and/or specificity of different PCR-based methods are dependent on genes and primer/probes selected. Although the primers and probes targeted conserved regions of the viral genome sequences, the variation of SARS-CoV-2 RNA sequence can produce mismatches in binding, leading to differences in the assay performance and potential false negative results. For this reason, a combination of targets is generally preferred for enhanced reliability.

However, taking into account both targets used in this study, a total of 54 SARS-CoV-2 positive samples, corresponding to 13.5%, were retrieved. Our discordant results cannot be ascribed to the uneven distribution of target RNA on surfaces, since the two amplification kits have been used on the same extracted sample. On the contrary, although samples have been correctly stored at −80 °C, the potential degradation of the viral RNA, especially at the very low concentration found in our samples, cannot be excluded. Finally, concerning the influence of matrix-associated inhibitors, it should be noted that the first kit of the multistep assay has been previously tested by us on different materials (plastic, metal, wood and paper) and the results reported in Casabianca et al.^26^ indicated that none of the four specific matrices affected RNA isolation, SARS-CoV-2 RNA detection or inhibited PCR, as revealed by the positive amplification signals of both the IPC and RdRp gene, reported in the above-mentioned article. The key element to explain the high number of discordant results should be sought, instead, in the very low amount of viral genomes in the environmental surfaces. This consideration is supported by Eibinger et al.^49^, who reported that SARS-CoV-2 RT-qPCR assays may show considerable variability, especially in samples with low viral RNA concentrations. FluA/B and RSV A/B were also detected with very low prevalence in the second and third time-periods, in line with their common seasonal distribution^5^.

Positive samples were mainly obtained both from classrooms in which students attended their lessons and from studying and dining tables, sampled immediately after lesson/lunch end and student exit. In contrast, samples from common areas (i.e., toilets, vending machine push-button panels) were always negative. These results could be explained by the fact that common areas’ cleaning and sanitizing is made more frequently than classrooms’, that are closed during lesson hours. Moreover, students and teachers reside longer in classrooms than in common areas, that are located in wide corridors and in places of passage, and the actual probability of contamination will increase with longer duration of staying in a room. Also, crowding level is different among those locations.

Finally, the third phase of the method consisted in the quantitative detection of hRpp40, a further single copy endogenous control for human DNA. This last step allowed the detection of human DNA in 9 additional samples, which were assigned as invalid in step 2. Thanks to the combined multistep analysis, the number of invalid samples was considerably reduced and the examination of the 400 samples led to a significant performance improvement in detecting viral RNA on environmental surfaces, going from 1 failed sample out of 13 to 1 out of 44, with a very high prevalence of valid results (98%).

The overall prevalence of SARS-CoV-2 in university surfaces reported by other authors is quite variable. Zhang et al.^40^, who carried out an investigation on a university campus located in the U.S. in 2020–2021, found a 1.4% prevalence of SARS-CoV-2 positive surface samples. Interestingly, these authors proved that the total case number on campus was significantly higher in weeks with positive environmental samples than in non-positive weeks (Point-biserial correlation, *p* = 0.001). Another study, conducted in 2023 at a university in Southern Italy^50^, reported a slightly higher prevalence of 5.4%, with a direct association between the average number of COVID-19 cases among university students in the seven days following sampling and the percentage of SARS-CoV-2 positive swabs on sampling day. A similar prevalence was detected in a previous study of our research group, carried out in Autumn-Winter 2021 in Central Italy^26^. However, a relevant point reported by De Giglio et al.^50^ was the greater contamination percentage found in the Spring–Summer period than in the Autumn, suggesting that an effective monitoring plan, to be implemented as an early warning system, could benefit from a more extensive sampling timespan. Finally, in the interval January-November 2022, Ali et al.^39^, sampling environmental surfaces in a large university campus in Florida, reported a very high SARS-CoV-2 viral RNA presence (90.7%), correlating with trends in community-level activity and case reports from the student health centre. In the present study, a percentage of positive samples corresponding to 16% resulted positive for the RNA of all the respiratory viruses tested, of which 14% for the sole SARS-CoV-2, only in the time interval late Autumn-early Winter. A comparison among the epidemiological data reported by different authors is nonetheless difficult, since data are strongly dependent on different factors, such as the epidemiological situation, virus circulation (including the various types and variants), mandatory use of personal protective equipment or other restrictions, along with the specific detection protocol used for surface sampling, extraction and amplification. However, since several students come daily or weekly from other cities as commuters, public data on the prevalence of diseases caused by these three pathogens in the University town could not mirror the epidemiological situation present at the Campus.

As outlined elsewhere^40^, the university environment represents a nonhealthcare but potentially risky setting, especially if considering lecture halls and exercise facilities^51^, in which the crowding or the peculiar activity performed, or also the sharing of tables and devices/equipment (i.e., PCs) determine a higher transmission potential. The model for quantitative microbial risk assessment, applied to estimate the probability of infection after exposure to SARS-CoV-2 by contact with contaminated surfaces, revealed a correlation between COVID-19 case numbers and the environmental positive samples^39,40,50^. Unfortunately, in our study the number of cases among the university population was not determined, but a similar correlation could be reasonably hypothesized and extended also to other respiratory viruses, such as those detected in this investigation. Therefore, the monitoring of surface environmental samples may represent a convenient indicator of potential infection spread in the community. The information obtained from high-touch surface monitoring could also allow to identify hotspot areas, requiring more frequent cleaning, sanitation and ventilation. Although the survival of viruses on surfaces is influenced by numerous factors, and the actual probability of transmission leading to infection via this route (fomite transmission) is likely not the primary one, the purpose of the environmental surveillance in this university setting had the primary objective of determining an early warning system to assess and mitigate the potential transmission risk in the specific environments in which samplings have been carried out. Indeed, the presence of viral genetic material on the surfaces reveals the transit and contact of infected individuals. Further objectives included the identification of potential transmission hotspots as priority areas for targeted intervention (i.e., sanitation) and the recognition of critical timeframes for enhanced prevention. Even if surface testing is a complement to preventive measures and environmental monitoring plans, it remains useful as part of the risk assessment to also ensure student and employees’ safety.

Results of this study allowed us to formulate specific prevention and control recommendations. We provided some evidence to suggest that there is an effect of enhancing class size to reduce the chance of finding positive samples. Small classrooms and inside them, student desks and teacher workstations, have been identified as priority areas for targeted interventions, such as daily cleaning and sanitation. Further specific measures may include the use of air sanitation devices providing microbial inactivation^52^. Moreover, at least in our investigation, the period of late Autumn (weeks 48–49) and the beginning of the new year (weeks 2–4) has been recognised as a critical timeframe, during which the application of the above-mentioned measures could offer the benefit of enhanced prevention. Moreover, the increment in Ct values recorded over the three time periods suggests a decrement in viral RNAs. The complexity of the academic environment, along with the several factors impacting on viral presence on surfaces (viral load, material, location, crowding, number of infected people, sanitation products, etc.), may influence sample recovery and test results. Therefore, proper method selection is a key element in environmental surveillance.

Our approach may represent a promising tool for non-healthcare public place as a university setting in surveilling fomites associated with respiratory viruses or other pathogen’s transmission. Although the 3-Step method is complex for routine monitoring, in this study, the combined use of the three assays allowed us to understand their usefulness and limitations, particularly in the detection of false negatives. Indeed, the complementary use of the 3 QCs allowed us to monitor the efficiency of the RNA extraction procedure and the effectiveness of the PCR reaction, and thus exclude the presence of false negatives with a sensitivity of 98%. However, testing all samples first, then performing QC only on the negative samples, could potentially reduce the number of steps and overall workload.

Finally, our goal was not to compare kits but to maximize detection while minimizing the number of invalid samples. The IPC, being an exogenous synthetic RNA fragment, may be unstable and consequently become un-amplifiable. On the contrary, an endogenous control (such as the endogenous human RNaseP of the second step method), is more reliable in revealing nucleic acid amplifiability. Finally, the introduction of a further endogenous target (Rpp40 endogenous control) offers the possibility to amplify a different human DNA fragment^33^, with possibly different amplification efficiency, thus complementing and integrating the so-called endogenous control system. The use of multiple (at least two) target viral sequences (i.e., RdRp and ORF plus N) is always highly recommended^53^ since some genetic fragments may contain sequence mutations that can affect detection reliability.

The operating protocol, which we describe in this work and extensively tested previously^26^, can be easily implemented using an automatic nucleic acid extractor. For example, the one present in our laboratories allows the extraction of 32 samples in 10–15 min with only a few manual steps by the operator, utilizing magnetic bead-based technology. Three nucleic acid extraction sessions (96 samples) can then be analyzed all together in a single real-time PCR plate (~ 2 h). Therefore, the analysis of approximately 100 samples can be done in one working day.

This medium-throughput protocol (extraction with a small automated extractor and semi-automated real-time PCR) does not require large spaces and sophisticated environmental surveillance laboratories.

Limitations of our study include the lack of correlation with epidemiologic data, the minimal detection of viruses other than SARS-CoV-2 and the absence of validation data due to the use of commercial assays with analytical sensitivity determined by synthetic RNA controls rather than environmental matrices.

## Conclusions

Environmental genomic surveillance represents a simple, cost-effective and non-invasive strategy to assess the exposure to respiratory viruses in confined environments and can complete other public health surveillance measures (i.e., contact tracing, clinical reports, and laboratory-based testing). Its potential as an additional source of real-life information to support evidence-based decisions on mitigation strategies should be considered, with particular concern of protection of fragile people attending educational environments. Our Surface 3-step PCR platform that simultaneously detects the three major circulating respiratory viruses using the approaches for procedures controls, due to its high sensitivity (98%) even with samples particularly difficult to test such as environmental ones ranging in a very low viral RNA levels, seems to be accurate and adequate for the purpose; however our research deserves further investigations with a greater number of samples.

## Supplementary Information

Below is the link to the electronic supplementary material.

Supplementary Material 1

## Data Availability

All data generated or analyzed during this study are included in this published article. Real-time PCR dataset relating to individual samples obtained from the developed multiplex assay (1st, 2nd, 3rd steps) are available from the corresponding author Anna Casabianca on reasonable request.
