# Supplementary material for: Simultaneous detection of respiratory virus RNA on environmental surfaces in a university setting by a sensitive Surface 3-Step PCR platform
Source: Sci Rep. 2025 Nov 3;15:38401. doi: 10.1038/s41598-025-22296-5 (PMC12583612; doi:10.1038/s41598-025-22296-5)
Supplement: Supplementary file 1 — Supplementary Material 1 [file 41598_2025_22296_MOESM1_ESM.docx]

**-Supplementary Information-**

**Simultaneous detection of respiratory virus RNA on environmental surfaces in a university setting by a sensitive Surface 3-Step PCR platform**

**Chiara Orlandi^1,2^, Giulia Amagliani**^3^**, Giorgio Brandi**^3^**, Asja Conti^3^, Giuditta Fiorella Schiavano**^4+^  **& Anna Casabianca^1,2,^** ^+^**^*^**

^1^Department of Biomolecular Sciences, Section of Biochemistry and Biotechnology, University of Urbino Carlo Bo, Fano (PU), 61032, Italy

^2^Laboratorio Covid, University of Urbino Carlo Bo, Fano (PU), 61032, Italy

^3^Department of Biomolecular Sciences, Unit of Hygiene, University of Urbino Carlo Bo, Urbino (PU), 61029, Italy

^4^Department of Humanities, University of Urbino Carlo Bo, Urbino (PU), 61029, Italy

^*^Corresponding author anna.casabianca@uniurb.it

^+^These authors share the co-senior authorship


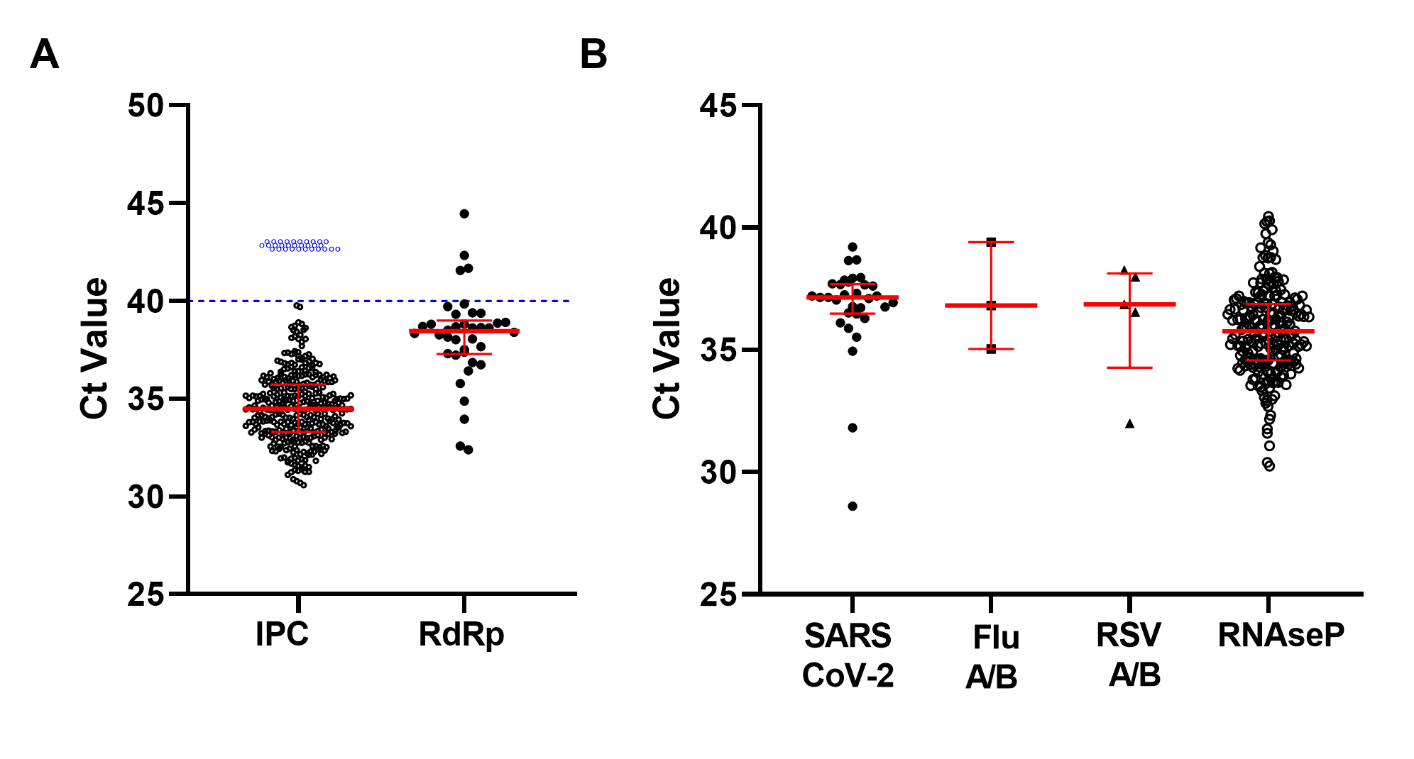
**Supplementary Fig. S1** A) Results from the first-step PCR in 400 environmental samples; 31 samples with an invalid result (Ct value >40) for the Internal Process Control (IPC) are represented as blue circles above the dashed line. Among 369 valid samples, 38 gave a positive amplification for RdRp gene of SARS-CoV-2 RNA. B) Results from the second-step PCR in 400 environmental samples; 32 samples gave a positive amplification for ORF1b/N genes of SARS-CoV-2, 3 for M1/NS2 genes of Flu A/B and 5 for M gene of RSV A/B. The endogenous control RNaseP is used to monitor the adequacy of the sample, the RNA extraction process, and the presence of PCR inhibitors. Red lines represent the median and 25th to 75th percentiles**.**

| **Step** | **Phase** | **Temperature and time** | **Cycles** |
| --- | --- | --- | --- |
| First step  SARS-CoV-2 Surface Kit | cDNA synthesis | 48°C 30 min | 1 X |
|  | Initial denaturation | 95°C 10 min | 1 X |
|  | Denaturation | 95°C 15 sec | 45 X |
|  | Annealing-extension | 60°C 30 sec |  |
|  | | | |
| Second step  COVID-FLU-RSV All-in-One RT PCR | cDNA synthesis | 48°C 20 min | 1 X |
|  | Initial denaturation | 95°C 2 min | 1 X |
|  | Denaturation | 95°C 10 sec | 42 X |
|  | Annealing-extension | 58°C 1 min |  |
|  | | | |
| Third step  SYBR Green qPCR assay  for Human Rpp40 | Initial denaturation | 95°C 10 min | 1 X |
|  | Denaturation | 95°C 15 sec | 40 X |
|  | Annealing-extension | 64°C 1 min |  |
|  | Dissociation curve | Automatic temperature increment to generate a melt curve | |

**Supplementary Table S1.** Thermal profile setting

| **Target** | **T1** | | **T2** | | **T3** | | **Overall** | |
| --- | --- | --- | --- | --- | --- | --- | --- | --- |
|  | **Ct** | **n** | **Ct** | **n** | **Ct** | **n** | **Ct** | **n** |
| IPC | 33.24 [32.27-34.23] | 123 | 34.53 [33.68-35.6] | 132 | 35.14 [34.45-36.33] | 114 | 34.48 [33.29-35.73] | 369 |
| SARS CoV-2 (RdRp) | 38.06 [36.8-38.84] | 17 | 38.45 [37.25-38.78] | 16 | 39.72 [38.83-43.39] | 5 | 38.45 [37.29-39.01] | 38 |
| SARS CoV-2 (ORF1b/N) | 37.14 [36.29-37.86] | 15 | 37.12 [36.52-37.27] | 14 | 37.65 [36.75-38.65] | 3 | 37.14 [36.48-37.69] | 32 |
| Flu A/B (M1/NS2) |  |  | 35.93 [35.04-36.82] | 2 | 39.41 [39.41-39.41] | 1 | 36.82 [35.04-39.41] | 3 |
| RSV A/B (M) |  |  | 34.99 [32-37.98] | 2 | 36.86 [36.54-38.26] | 3 | 36.86 [34.27-38.12] | 5 |
| Human Rnase P | 35.9 [34.63-36.94] | 86 | 35.4 [34.44-36.78] | 65 | 35.92 [34.57-36.88] | 77 | 35.76 [34.57-36.88] | 228 |
| Human Rpp40 |  |  | 32.73 [31.46-33.33] | 3 | 31.76 [30.89-33.40] | 6 | 31.88 [31.22-33.20] | 9 |

**Supplementary Table S2.** Ct values, as median [IQR], for the respiratory viruses, Internal Process Control (IPC) and human endogenous controls.

| **Step** | **Target** | **Ct** |
| --- | --- | --- |
| First step  SARS-CoV-2 Surface Kit | IPC | 28.96 [28.88-29.65] |
|  | SARS CoV-2 (RdRp) | 32.30 [32.01-32.91] |
| Second step  COVID-FLU-RSV All-in-One RT PCR | SARS CoV-2 (ORF1b/N) | 22.21 [21.97-23.01] |
|  | Flu A/B (M1/NS2) | 26.41 [26.18-27.01] |
|  | RSV A/B (M) | 25.97 [25.62-26.03] |
|  | Human Rnase P | 21.42 [20.87-22.09] |
| Third step  SYBR Green qPCR assay  for Human Rpp40 | Human Rpp40 (copy no.) |  |
|  | 10^5 | 16.23 [16.07-16.39] |
|  | 10^4 | 19.93 [19.73-20.13] |
|  | 10^3 | 23.15 [22.68-23.61] |
|  | 10^2 | 26.56 [26.28-26.84] |
|  | 10 | 29.61 [29.43-30.05 |
|  | 2 | 31.88 [31.62-32.16] |

**Supplementary Table S3**. Ct values, as median [IQR], for positive PCR control of Internal Process Control (IPC), respiratory viruses and human endogenous controls.

|  | **Sample ID** | First-step PCR  (RdRp gene) | Second-step PCR PCR  (ORF1b/N genes) | **Time point** |
| --- | --- | --- | --- | --- |
| 1 | 30 | 36.85 | Undetermined | T1 |
| 2 | 34 | 37.66 | Undetermined | T1 |
| 3 | 39 | 38.06 | Undetermined | T1 |
| 4 | 41 | 38.33 | Undetermined | T1 |
| 5 | 54 | 41.57 | Undetermined | T1 |
| 6 | 55 | 38.02 | Undetermined | T1 |
| 7 | 82 | 32.39 | Undetermined | T1 |
| 8 | 86 | 38.81 | Undetermined | T1 |
| 9 | 99 | 38.87 | Undetermined | T1 |
| 10 | 101 | 38.61 | Undetermined | T1 |
| 11 | 146 | 38.70 | Undetermined | T2 |
| 12 | 152 | 38.15 | Undetermined | T2 |
| 13 | 169 | 37.23 | Undetermined | T2 |
| 14 | 171 | 36.41 | Undetermined | T2 |
| 15 | 181 | 39.32 | Undetermined | T2 |
| 16 | 182 | 39.85 | Undetermined | T2 |
| 17 | 253 | 38.90 | Undetermined | T2 |
| 18 | 254 | 38.67 | Undetermined | T2 |
| 19 | 257 | 38.61 | Undetermined | T2 |
| 20 | 299 | 44.45 | Undetermined | T3 |
| 21 | 303 | 38.27 | Undetermined | T3 |
| 22 | 395 | 39.72 | Undetermined | T3 |
| **Median [IQR]** | | **38.61 [37.93-39-01]** |  |  |
|  | | | | |
| 1 | 2 | Undetermined | 36.10 | T1 |
| 2 | 25 | Undetermined | 36.47 | T1 |
| 3 | 37 | Undetermined | 37.97 | T1 |
| 4 | 42 | Undetermined | 37.61 | T1 |
| 5 | 77 | Undetermined | 37.93 | T1 |
| 6 | 97 | Undetermined | 37.67 | T1 |
| 7 | 100 | Undetermined | 37.86 | T1 |
| 8 | 110 | Undetermined | 38.68 | T1 |
| 9 | 217 | Undetermined | 36.73 | T2 |
| 10 | 224 | Undetermined | 37.10 | T2 |
| 11 | 226 | Undetermined | 36.78 | T2 |
| 12 | 227 | Undetermined | 37.20 | T2 |
| 13 | 256 | Undetermined | 37.25 | T2 |
| 14 | 262 | Undetermined | 37.32 | T2 |
| 15 | 268 | Undetermined | 39.21 | T2 |
| 16 | 292 | Undetermined | 38.65 | T3 |
| **Median [IQR]** | |  | **37.47 [36.86-37.96]** |  |
|  | | | | |
| 1 | 29 | 41.68 | 37.14 | T1 |
| 2 | 33 | 37.38 | 37.70 | T1 |
| 3 | 36 | 38.64 | 36.29 | T1 |
| 4 | 43 | 39.37 | 36.51 | T1 |
| 5 | 89 | 33.96 | 34.95 | T1 |
| 6 | 93 | 36.75 | 36.93 | T1 |
| 7 | 96 | 34.87 | 31.82 | T1 |
| 8 | 165 | 38.40 | 37.15 | T2 |
| 9 | 207 | 35.77 | 37.77 | T2 |
| 10 | 213 | 38.81 | 35.51 | T2 |
| 11 | 233 | 38.51 | 37.04 | T2 |
| 12 | 235 | 32.58 | 28.60 | T2 |
| 13 | 252 | 37.52 | 35.88 | T2 |
| 14 | 267 | 37.31 | 37.20 | T2 |
| 15 | 307 | 39.38 | 37.65 | T3 |
| 16 | 323 | 42.320 | 36.751 | T3 |
| **Median [IQR]** | | **37.96 [36.02-39.23]** | **36.84 [35.6-37.19]** |  |

**Supplementary Table S4.** Comparison of SARS CoV-2 Ct values for 54 positive environmental samples
